# Revascularization of a Necrotic Femoral Head in Severely Slipped Capital Femoral Epiphysis With a Modified Dunn Procedure: A Case Report

**DOI:** 10.7759/cureus.53530

**Published:** 2024-02-03

**Authors:** Junya Shimizu, Hiroki Fujita, Kenji Tateda, Ima Kosukegawa, Atsushi Teramoto

**Affiliations:** 1 Department of Orthopedic Surgery, Sapporo Medical University, Sapporo, JPN; 2 Department of Orthopedic Surgery, Hokkaido Medical Center for Child Health and Rehabilitation, Sapporo, JPN; 3 Department of Orthopedic Surgery, Sapporo Kojinkai Memorial Hospital, Sapporo, JPN; 4 Department of Orthopedic Surgery, School of Medicine, Sapporo Medical University, Sapporo, JPN

**Keywords:** child, non-weight bearing, restored blood flow, modified dunn procedure, slipped capital femoral epiphysis

## Abstract

Avascular necrosis, a serious slipped capital femoral epiphysis (SCFE) complication, is difficult to treat.

We report a rare case of revascularization of the necrotic femoral head in a 12-year-old male patient with a severe SCFE (posterior tilting angle, 87°). We performed the modified Dunn procedure (MDP), followed by long-term unloading therapy. Blood flow to the epiphysis had partially resumed 2.3 years postoperatively. At the final 4.5-year follow-up, blood flow had been restored, leading to epiphyseal closure without significant femoral head deformity or hip pain. The patient could walk unassisted, with a flexion range of 120°.

These findings support the use of the MDP with long-term unloading therapy as a potential treatment option for severe SCFE.

## Introduction

Avascular necrosis (AVN) is a catastrophic complication of slipped capital femoral epiphysis (SCFE). Once the necrotic epiphysis collapses, the femoral head deformity becomes irreparable, potentially resulting in significant disability. SCFE, in which the capital epiphysis is usually displaced both posteriorly and inferiorly, occurs in approximately 8-10 individuals per 100,000 in Western countries [1]. The incidence of SCFE has been increasing. In Japan, the average annual incidence between 1997 and 1999 was 2.2 individuals per 100,000, which is five times higher than that in the eastern half of Japan in 1976 [2]. Some other studies also reported increases in the incidence of SCFE.

SCFE severity is classified as mild (posterior tilting angle [PTA] < 30°), moderate (PTA 30°-50°), or severe (PTA > 50°). Severe SCFE has poor outcomes. Furthermore, the risk of AVN is higher in patients with unstable and acute SCFE compared with those with mild SCFE [3]. Once AVN occurs following SCFE, no effective treatment has been reported to date.

To the best of our knowledge, there are no reports of revascularization after AVN following SCFE. In this report, we present a case of improved blood flow in a 12-year-old boy with AVN following SCFE, treated with the modified Dunn procedure (MDP) followed by long-term non-weight-bearing (NWB) therapy.

## Case presentation

Current medical status

**Figure 1:**
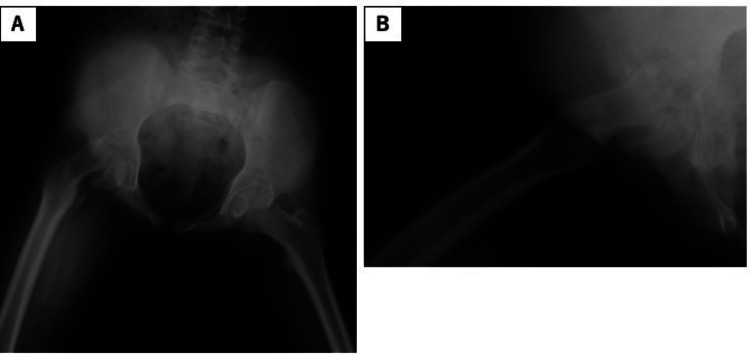
Radiograph (taken at our hospital) revealing more severe slippage, manifesting as a head-shaft angle of 91° and the posterior tilting angle of 87° (arrow). (A) Anteroposterior view; (B) lateral view.

**Figure 2:**
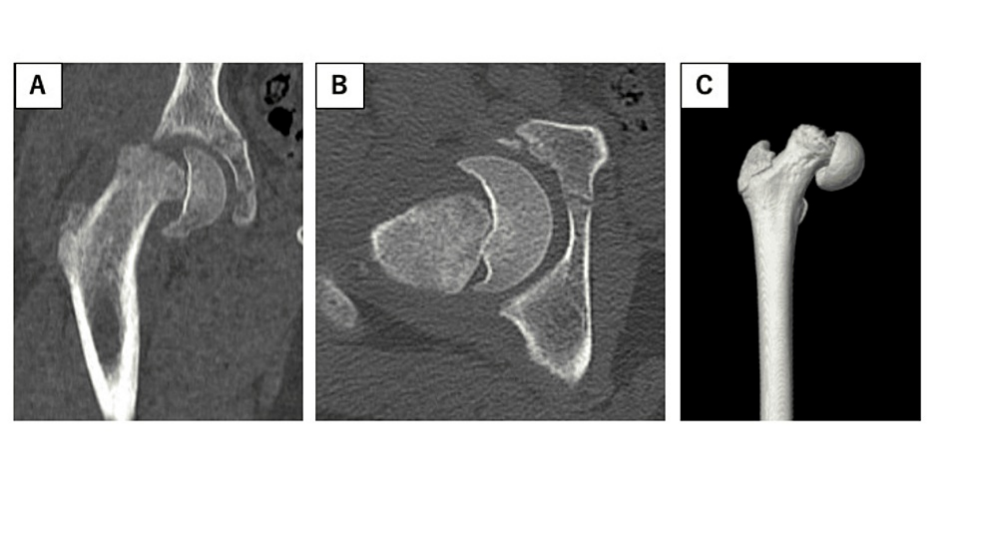
Computed tomography revealing 63° of internal rotation, with 15° of posterior dislocation relative to the unaffected side(arrow). (A) Coronal view; (B) axial view; (C) three-dimensional view.

**Figure 3:**
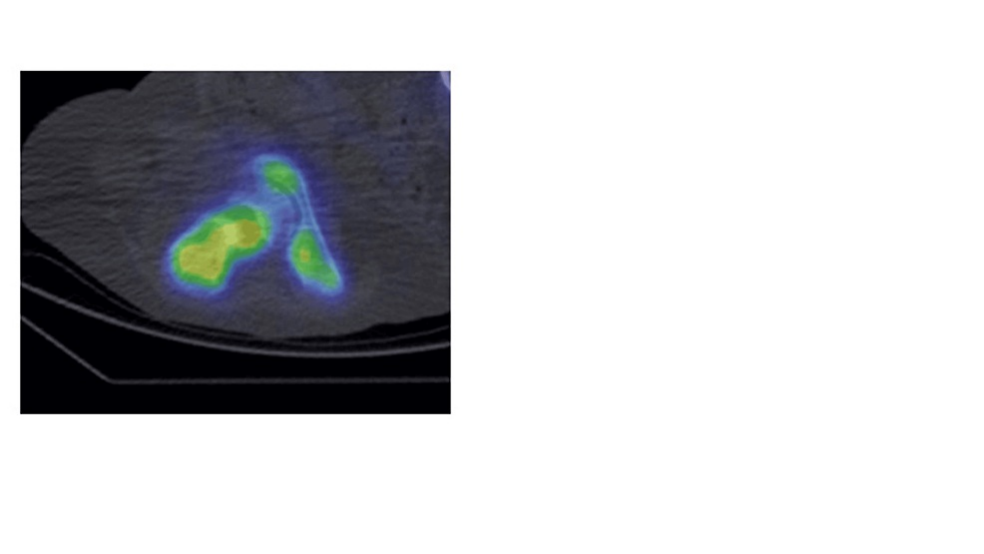
SPECT/CT depicting the absence of uptake in the right femoral head extending into the right femoral epiphysis (arrow). SPECT, single-photon emission computed tomography

A 12-year-old boy noticed right-hip pain after playing basketball. One week later, he was taken to a local hospital due to a fall at school, rendering him unable to walk due to severe right-hip pain. He was diagnosed with right SCFE and referred to our hospital two weeks later. Upon admission, his height measured 140 cm, weight was 45 kg, and the body mass index (BMI) was calculated at 22.9 kg/m². He had no obvious endocrine disorders such as hypogonadism. Right-hip pain during flexion was noted. Tenderness was present in the right Scarpa’s triangle; Drehmann’s sign was noted on the right side. Plain radiographs revealed a slipped femoral head with Southwick's anteroposterior (AP) angle (shaft epiphysis proximal femoral AP angle) of 91° (normal range 144°-155° [6]) and posterior tilting angle (PTA) of 87° on the affected side (Figures 1A-1B). Plain computed tomography (CT) revealed 63° of internal rotation and 15° of posterior dislocation on the right side (Figures 2A-2C). Single-photon emission CT/CT (SPECT/CT) revealed no uptake into the epiphysis of the right femoral head (Figure 3). The patient was diagnosed with severe unstable SCFE (PTA 87°). 

**Figure 4:**
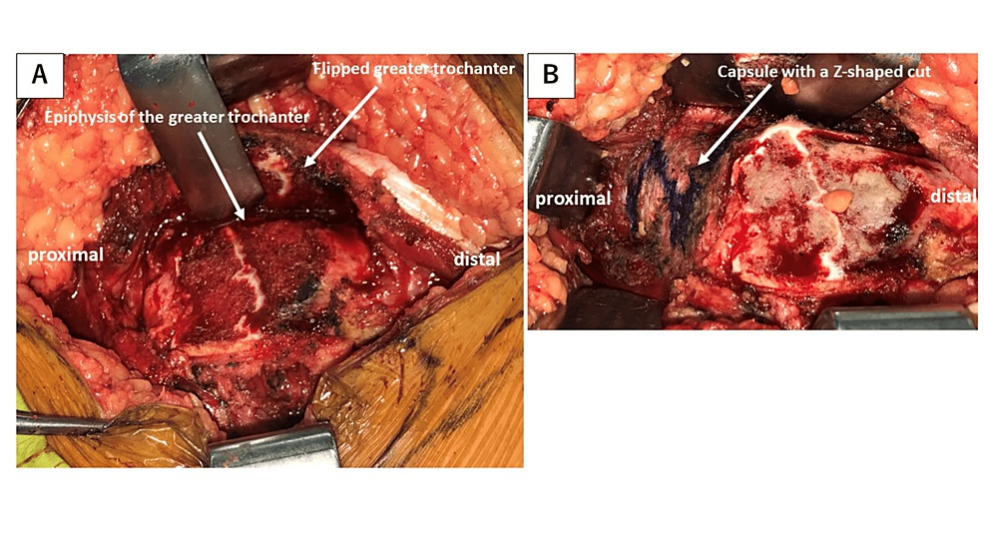
Intraoperative photograph showing trimming of the epiphysis (arrow); this was followed by screw fixation.

Based on the severe displacement, we performed the MDP. The patient was placed in the lateral decubitus position. A straight lateral incision was made. The tensor fascia lata was split anteriorly to the gluteus maximus. The greater trochanter was osteotomized via flip osteotomy, and the capsule was incised in a Z shape to expose the femoral head (Figure 4). The head was internally displaced, and the slipped femoral head was unstable. The periosteum containing the nutritional blood vessels was cut from the lateral to the posterior side, and a Kirschner steel wire was inserted into the epiphysis. No blood flow was observed in the necrotic femoral head. Following hip dislocation with the release of the ligamentous teres, the epiphysis was trimmed. After the epiphysis was curetted to remove the physeal cartilage, the epiphysis was reduced. The epiphysis was fixed with a 6.5 mm cannulated screw. The capsule was closed. Thereafter, the trochanteric fragments were replaced and fixed with two screws. Postoperative contrast-enhanced magnetic resonance imaging (MRI) revealed no blood flow to the epiphysis. The patient was instructed to use a wheelchair and prohibited from bearing weight on the affected side.

**Figure 5:**
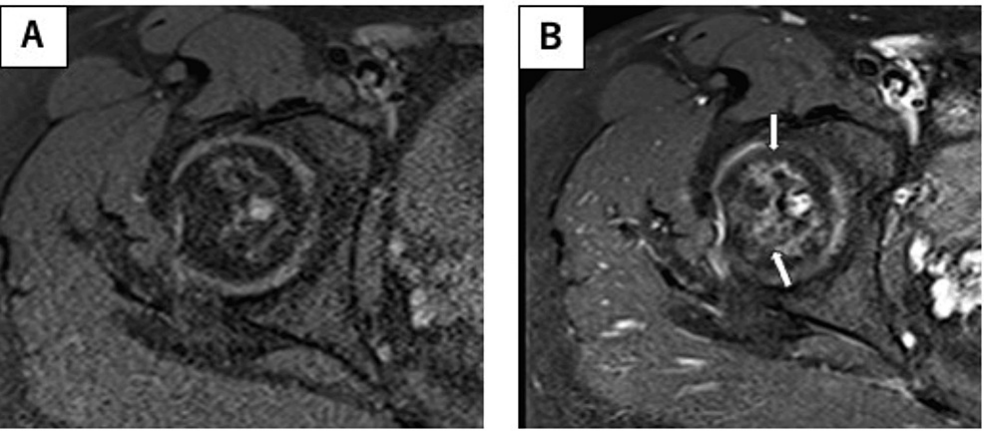
Gadolinium-enhanced magnetic resonance image taken 2.3 years postoperatively, revealing a partial contrast effect at the epiphysis (arrow). (A) Plain T1WI axial view; (B) gadolinium-enhanced T1WI axial view.

**Figure 6:**
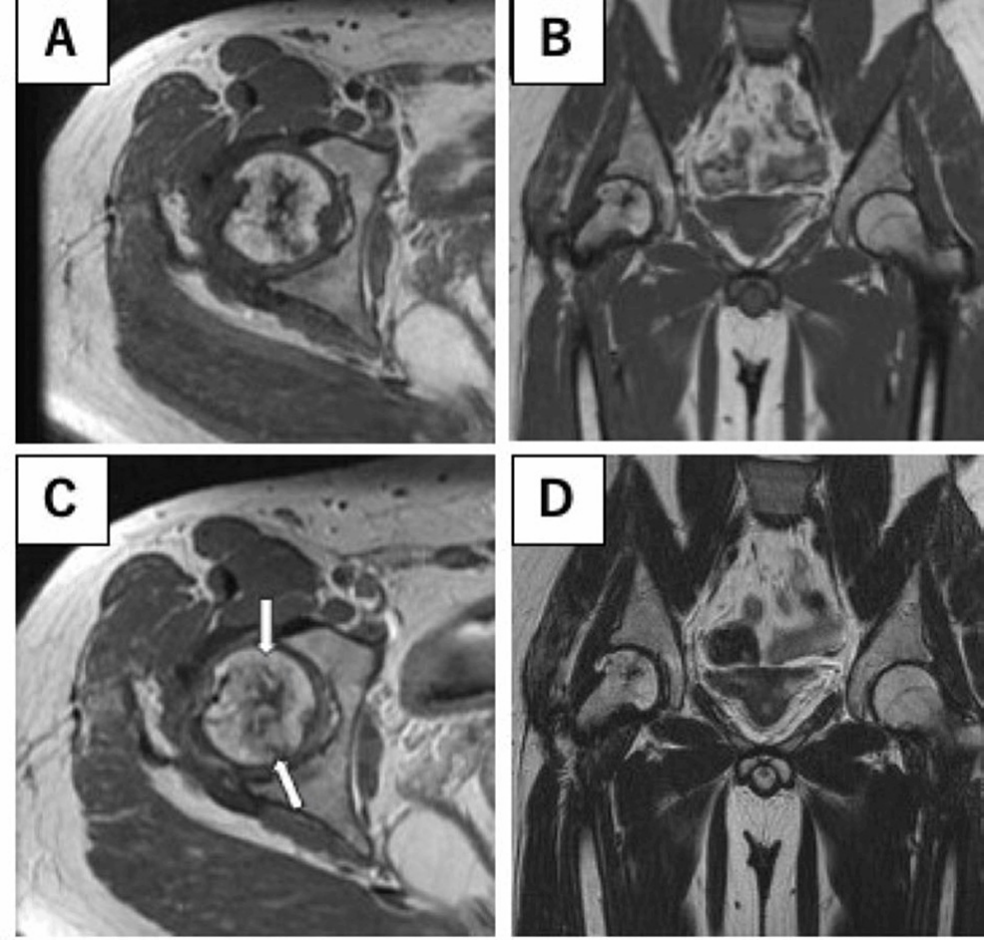
Magnetic resonance image taken 3.6 years postoperatively, revealing a greater contrast effect at the epiphysis (arrow). (A) Plain T1WI axial view; (B) plain T1WI coronal view; (C) gadolinium-enhanced T1WI axial view; (D) plain T2WI coronal view.

**Figure 7:**
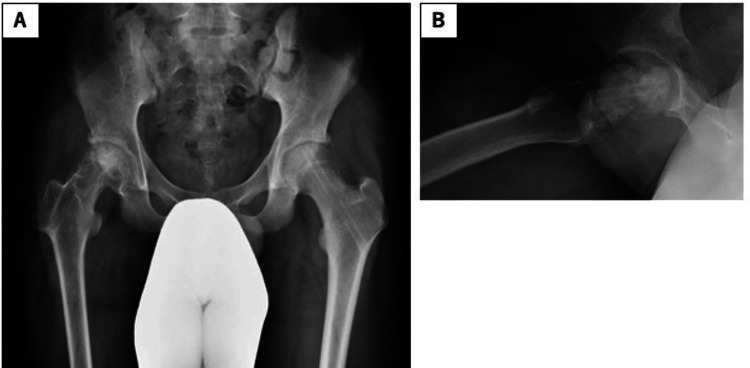
Plain radiographs at final follow-up (4.5 years postoperatively) revealing epiphyseal closure, without severe femoral head deformity. (A) Anteroposterior view; (B) lateral view.

MRI performed 2.3 years postoperatively showed a partial contrast effect at the epiphysis (Figure 5). Subsequently, an MRI conducted 3.6 years postoperatively revealed an increased contrast effect (Figure 6). Further assessment revealed improved blood flow to the epiphysis. Accordingly, partial loading was implemented, gradually increasing to full loading over three months. Plain radiographs at the final follow-up (4.5 years postoperatively) revealed epiphyseal closure without severe femoral head deformity, despite some subchondral cyst formations (Figure 7). No hip pain was observed, and the patient could walk unassisted, with 120° flexion.

## Discussion

Surgical treatment is difficult for severe SCFE. Treatment options for severe SCFE include in situ pinning (ISP) and the MDP. ISP has a few complications and relatively good long-term results. However, screw insertion is technically demanding because of the severe posterior displacement, and impingement may occur in cases of poor remodeling. AVN rate was reported to be 4%-55% after ISP for unstable SCFE, as presented in Table 1 [4-6]. Ramachandran et al. [5] reported a very high incidence of AVN after ISP but did not perform capsulotomy in all cases, suggesting that capsulotomy was necessary when performing ISP. The MDP is a good option for treating severe SCFE. The MDP was first described by Ganz et al. [7] in 2001 using surgical dislocation, which was a modification of the original Dunn method. The MDP could reduce the risk of AVN. In 2007, Leunig et al. [8] reported performing the MDP to open reduction and fixation for moderate and severe SCFE, after which no patients had AVN after the MDP. Recently, the MDP has been spread gradually for moderate to severe SCFE due to low incidences of AVN [9-14]. Most importantly, the MDP enables intraoperative evaluation of epiphyseal perfusion [15]. During the procedure, the anatomical reduction can be performed while preserving the blood supply. Zaltz et al. [16] reported that they reviewed 397 patients in 15 articles with unstable SCFE and reported the rate of AVN was 23.9% without mentioning the degree of slippage. However, the MDP does not necessarily prevent AVN; 6%-26% of patients treated with the MDP for unstable SCFE develop AVN [12]. In a multicenter study, up to 26% of patients developed AVN following the MDP for unstable SCFE [10]. Salvage surgery was performed in 18 out of 20 patients with AVN after SCFE; in the remaining two patients, lateral shelf acetabuloplasty was performed. Furthermore, there remains a lack of consensus on the appropriate timing for the MDP. Novais et al. [15] could not determine the optimal timing for surgery. 

**Table 1:** Previous reports of AVN rate following MDP for unstable SCFE. MDP, modified Dunn procedure; ISP, in situ pinning; AVN, avascular necrosis; SCFE, slipped capital femoral epiphysis

Author	Year	Surgical method	Capsulotomy	Hips	Follow-up period (years)	AVN rate (%)
Sankar et al.［9］	2013	MDP	＋	27	2 (1-4)	26
Madan et al. [10]	2013	MDP	＋	28 (17 hips: unstable)	3 (2-7)	14
Persinger et al. [11]	2018	MDP	＋	31	2 (0-7)	6
Novais et al. [12]	2019	MDP	＋	27	2 (2-3)	26
Davis et al.［13］	2019	MDP	＋	31	2.3	6
Gordon et al.［4］	2002	ISP	6, percutaneous, 4 open	16	2	12
Ramachandran et al. [5］	2007	ISP	None	22	3	55
Chen et al. [6]	2009	ISP	16, percutaneous, 5 open	30	5	4

After SCFE, AVN is the most serious complication in children and has the potential to result in a devastating prognosis. The management of AVN after SCFE remains difficult. Although no guidelines for this are available, Nakashima et al. [17] reported that transchanteric rotational osteotomy was an effective salvage surgery, which was developed for adult AVN by Sugioka in 1978 [18], for AVN after unstable SCFE. Regarding AVN treatment, the primary objective is to prevent femoral head collapse and deformity. Atsumi et al. [19] reported that during the process of revascularization, subchondral fractures occur because of weakness of the affected head and mechanical stress. Kim et al. [20] reported that localized NWB therapy decreased deformity following osteonecrosis of the femoral head and increased the rates of revascularization and resorption of the necrotic epiphysis. To preserve the round shape of the necrotic femoral head, we elected to perform a long-term NWB treatment until epiphysial closure, followed by confirming blood flow to the epiphysis using contrast-enhanced MRI.

The slippage observed in this 12-year-old patient was severe, with a PTA of 87°. Two weeks after the injury, bone scintigraphy revealed no blood flow to the epiphysis, which indicated reduced bone turnover on the femoral capital epiphysis. Therefore, we chose to perform the MDP, because we expected intramedullary blood flow to accompany epiphyseal closure. Here, blood flow to the epiphysis had partially resumed 2.3 years postoperatively and had improved by the final follow-up at 3.5 years. The closure of the epiphyseal line was probably facilitated by intramedullary blood flow from the distal femur. There are a few reports of resumed blood flow to necrotic epiphyses. In one case, blood flow from the center to the surface of the epiphysis resumed one year after traumatic anterior dislocation of the hip; revascularization from the periphery to the center (in contrast to what occurred in our case), occurred in another case [20].

The optimal test for evaluating femoral head blood flow remains unclear; postoperative contrast-enhanced MRI is not commonly employed to assess revascularization. However, contrast-enhanced MRI is less invasive and minimizes radiation exposure. Therefore, it is useful for blood flow assessments in patients with SCFE. In this patient, it was possible to assess the revascularization of the femoral head after SCFE without performing bone scintigraphy. Thus, contrast-enhanced MRI is an effective postoperative evaluation tool.

## Conclusions

Although necrosis of the femoral head was observed during surgery, blood flow to the femoral head improved after 3.5 years of long-term postoperative unloading therapy, and the patient was able to walk unassisted, without crushing the femoral head. The MDP with long-term unloading therapy, therefore, might provide a potential option for treating severe SCFE.
